# Distribution Pattern, Ecological Determinants and Conservation Gaps of Model‐Predicted Relative Probability of Occurrence Zones for the Tufted Deer (*Elaphodus cephalophus*) in China

**DOI:** 10.1002/ece3.72850

**Published:** 2026-01-08

**Authors:** Yuangang Yang, Peng Luo, Yu Zhao, Hua Li, Yufang Yang, Mengyao Li, Tongzuo Zhang, Feng Jiang, Zhangqiang You

**Affiliations:** ^1^ School of Life Sciences Mianyang Normal University Mianyang China; ^2^ Forest Ecology and Conservation in the Upper Reaches of the Yangtze River Key Laboratory of Sichuan Province Mianyang China; ^3^ Key Laboratory of Research and Conservation of Biological Diversity in Minshan Mountain of National Park of Giant Pandas at Mianyang Normal University of Sichuan Province Mianyang China; ^4^ School of Biological and Pharmaceutical Sciences Mianyang Normal University Mianyang China; ^5^ Qinghai Provincial Key Laboratory of Animal Ecological Genomics, Northwest Institute of Plateau Biology Chinese Academy of Sciences Xining China

**Keywords:** conservation gap, ecological determinant, MaxEnt model, model‐predicted relative probability of occurrence, the tufted deer

## Abstract

The tufted deer ( *Elaphodus cephalophus* ), a rare ungulate species endemic to China, faces mounting conservation concerns due to habitat fragmentation, climate change, and historical overhunting. However, its current patterns of model‐predicted relative probability of occurrence and the environmental associations of its distribution remain poorly understood. In this study, we used 429 occurrence points and 28 environmental variables, refined to 11 key predictors, to predict the species' relative probability of occurrence across China using an optimized MaxEnt model. The model performed robustly, identifying six dominant environmental factors—temperature annual range, annual precipitation, mean temperature of the coldest quarter, slope, vegetation fractional cover, and human footprint index—that collectively contributed 91.6% to the model‐predicted relative probability of occurrence. Model outputs indicated that the relative probability of occurrence was associated with moderate temperature variation (25.3°C–30.4°C), bimodal precipitation patterns (725–1324 mm and 1651–1898 mm), and cooler winter temperatures (−2.0°C–9.9°C), typically found in mountainous regions. Model‐based analyses revealed that zones with moderate‐to‐high model‐predicted relative probability of occurrence are concentrated in eight provinces, with Sichuan, Guizhou, and Yunnan contributing the largest zones. Despite these extensive zones with model‐predicted relative probability of occurrence, our GAP analysis showed that 93.98% of them lie outside current protected zones overlapping with model‐predicted relative probability of occurrence areas, indicating substantial conservation gaps. Even in core provinces such as Sichuan and Guizhou, only a small fraction (≤ 10.84%) of the zones with high model‐predicted relative probability of occurrence are protected. These findings highlight the urgent need to re‐evaluate and expand protected zones networks to include zones with high model‐predicted relative probability of occurrence for tufted deer. Our study provided essential ecological insights and spatial data to guide habitat conservation, functional zoning, and long‐term management strategies for tufted deer populations in China.

## Graphical abstract

This study analyzed the habitat suitability of the tufted deer, a rare and endemic ungulate in China, using the MaxEnt model and found that suitable habitats are mainly concentrated in Sichuan, Guizhou, and Yunnan provinces. However, the majority of these areas are not covered by existing protected areas, highlighting the urgent need to expand conservation efforts. The findings provide crucial data and guidance for the protection and habitat management of tufted deer populations in China.

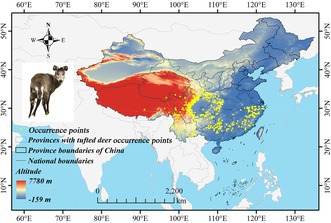

## 1 Introduction

The biodiversity crisis, starkly exemplified by wildlife declines, constitutes one of the most severe challenges facing humanity in the 21st century. Current estimates indicate that vertebrate extinction rates are more than 100 times higher than natural background rates, suggesting Earth is undergoing its sixth mass extinction event (McCallum 2015; Pringle 2017). Between 1970 and 2020, monitored global wildlife populations experienced an average decline of 73% (WWF 2024). This trend varies across ecosystems, with average population decreases of 85% for freshwater species, 69% for terrestrial species, and 56% for marine species. Biodiversity loss is occurring globally, with particularly acute reductions observed in Latin America and the Caribbean (95%), Africa (76%), and the Asia‐Pacific region (60%) (WWF 2024). To date, the IUCN Red List has assessed over 166,000 species, with more than 46,330 classified as threatened with extinction. This includes 41% of amphibians, 26% of mammals, 12% of birds, and 37% of sharks and rays (IUCN 2025). Biodiversity conservation is crucial for human well‐being and is a high priority for governments and international organizations worldwide. Reducing the rate of biodiversity loss is a shared global objective (Johnson et al. 2017), and research in wildlife conservation biology, particularly for rare species, is fundamental to effective biodiversity protection.

Given that the protection of a species is inextricably linked to the preservation of its living space, habitat modeling based on species distribution models (SDMs) has become a widely used tool in modern conservation management and strategic planning (Jiang et al. 2025). This approach relies on analyzing environmental associations with species occurrence, including potential refuges and key factors correlated with its survival and reproduction. The goal is to identify geographical zones with higher model‐predicted relative probability of occurrence, thus providing a spatial framework for conservation efforts (Guillera‐Arroita et al. 2015; Zahoor et al. 2021; Shangguan et al. 2024). Furthermore, identifying conservation gaps for rare and endangered species is of paramount importance (Scott et al. 1993). Habitat fragmentation from human activities like deforestation and urbanization, combined with climate‐driven shifts in species distribution, increasingly isolates wildlife populations. These isolated populations are more vulnerable to extinction due to reduced genetic diversity, increased inbreeding, and limited dispersal opportunities (Sterling et al. 2012; Vasconcellos et al. 2019). Therefore, a holistic habitat conservation strategy must include identifying these gaps, optimizing the boundaries of existing protected areas, and refining their internal functional zonation (Zhao et al. 2024).

Species Distribution Models (SDMs) have emerged as a widely utilized approach for predicting species' relative probability of occurrence (Guisan and Thuiller 2005). SDMs quantify the correlation between environmental factors and the distribution of plant and animal species. This empirically derived environmental profile can be used to measure the importance of specific factors, predict species distribution across unsampled areas, and examine the ecological consequences of environmental change (Miller 2010). Importantly, SDM outputs approximate the realized niche and reflect statistical associations with current occurrence records rather than direct measures of habitat quality or “optimal” conditions (Pulliam 2000; Lovell et al. 2023). One such SDM, the MaxEnt model, has been extensively applied to predict and map zones with higher model‐predicted relative probability of occurrence for a wide range of taxa, including birds (Zhou et al. 2025), ungulates (Evcin et al. 2019), carnivores (Yang, Deng, et al. 2025), and fish (McGarvey et al. 2021). For instance, Tang et al. (2022) used the MaxEnt model to analyze the effects of different climate scenarios on the model‐predicted potential distribution of the endangered white‐lipped deer ( *Cervus albirostris* ). Their results indicate that amid global warming, the total zones with high predicted relative probability of occurrence for the white‐lipped deer are projected to decline, accompanied by significant shifts in its population distribution. The finding that a large portion (73.08%) of these zones with high model‐predicted relative probability of occurrence lies outside the existing protected zones network underscores the urgent need to strengthen conservation efforts for this species (Tang et al. 2022).

The tufted deer ( *Elaphodus cephalophus* ) is a small, relatively primitive ungulate belonging to the order Artiodactyla, family Cervidae, and the monotypic genus *Elaphodus*. As a species of significant conservation interest, it is primarily distributed within the mountainous regions of China, with smaller populations extending into northern Myanmar (Harris and Jiang 2015). Morphologically, it is distinguished by a prominent blackish‐brown tuft of hair on its forehead, which gives the species its common name, and by the elongated, tusk‐like upper canines in males.

Within China, the species' range historically encompasses 16 provinces and autonomous regions, with major populations in Sichuan, Gansu, Qinghai, Anhui, and Hunan (Wei 2022). It typically occupies moist, high‐altitude forests and adjacent shrublands, often in rugged and topographically complex terrain. Despite its broad distribution, robust estimates of its total population size or current trend are unavailable. This data deficiency is partly attributable to the deer's elusive nature and the logistical challenges of conducting surveys in its remote habitat. A widely cited but speculative estimate by Sheng et al. (1998) suggested a population of 300,000–500,000 individuals in China, a figure that is now over two decades old and requires contemporary validation. Historically, the tufted deer has faced significant anthropogenic pressure, most notably from intensive poaching. Commercial fur trade data from the late 1970s to the early 1980s indicated an annual harvest of approximately 100,000 individuals across 12 provinces. This harvest was heavily concentrated in key regions, with an estimated 36,000 taken annually in Sichuan, 27,000 in Hunan, and 14,000 in Guizhou, followed by substantial numbers in other provinces. Although recent harvest data are lacking, the scale of this past exploitation, coupled with ongoing pressures on large mammals in China, strongly suggests a population decline (Sheng and Lu 1985; Harris and Jiang 2015). In recognition of these threats, the tufted deer is now a Level II key protected animal in China, affording it the highest level of legal protection. Furthermore, it is assessed as Vulnerable (VU) on China's Red List of Biodiversity, reflecting its high risk of extinction at the national level (Jiang 2021). In recent years, the tufted deer has become a subject of growing scientific inquiry aimed at building a foundation for its evidence‐based conservation. Scholarly investigations have begun to elucidate key aspects of its biology, including its morphology (Wang et al. 2007), feeding ecology (Yue 2014), behavior (Liu et al. 2021), and population genetics (Pang et al. 2008; Sun et al. 2016). Nevertheless, critical knowledge gaps persist. A comprehensive understanding of its model‐predicted relative probability of occurrence and the environmental factors associated with higher probabilities of occurrence across China remains largely unknown. Addressing this gap is fundamental for developing effective, spatially explicit conservation strategies.

Here, we selected the tufted deer in China as the focal species and utilized the MaxEnt model and ArcGIS to address two key objectives: (i) identifying the primary environmental factors associated with the model‐predicted relative probability of occurrence for the tufted deer in China, (ii) identifying the spatial distribution patterns of zones with higher model‐predicted relative probability of occurrence, and the conservation gaps. Through a comprehensive assessment of model outputs representing relative probability of occurrence, this study aimed to provide critical ecological data to inform and support conservation and management strategies for the tufted deer across its range within China.

## 2 Materials and Methods

### 2.1 Collection and Organization of Tufted Deer Distribution Site Data in China

**FIGURE 1:**
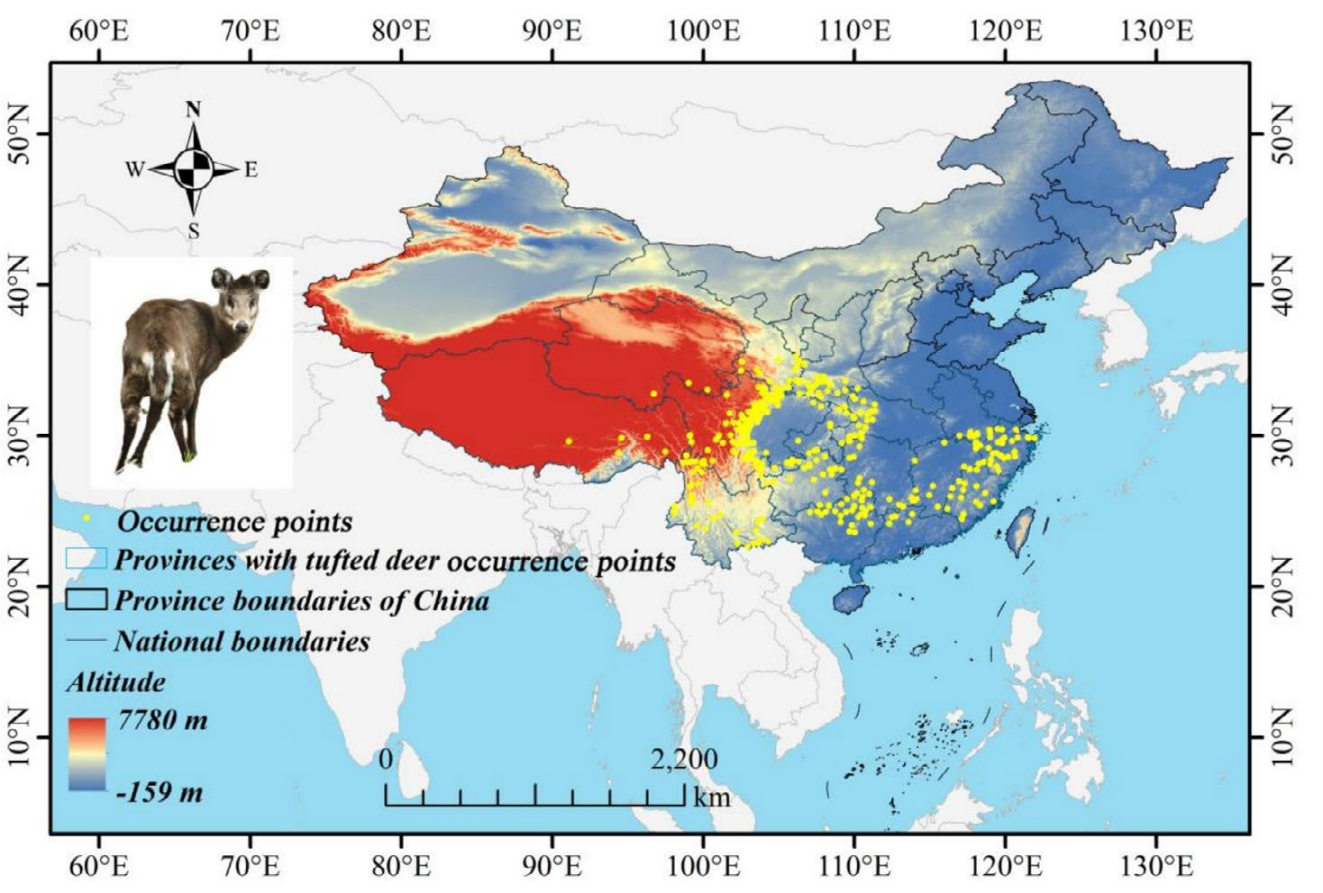
Occurrence points of the tufted deer in China; Photograph of a tufted deer taken in the Wanglang Area of Giant Panda National Park (Wanglang Nature Reserve) by Chunping Luo (GS 20240650).

In this study, occurrence points for the tufted deer (*Tufted deer*) in China were compiled from multiple sources including China's Red List of Biodiversity (CRLB, Jiang 2021), the Global Biodiversity Information Facility (GBIF, http://www.gbif.org), and the National Herbarium Resource Centre (NSII, http://www.nsii.org.cn). To ensure the plausibility of occurrence records, each point was visually inspected using high‐resolution satellite imagery from Google Earth (https://earth.google.com/web). The surrounding habitat within a ~200 m radius of each point was examined to verify whether it matched the known ecological preferences of the tufted deer—primarily forest, shrubland, and montane meadow habitats—and to exclude obviously unsuitable environments such as urban areas, croplands, large water bodies. Records failing to meet these criteria were removed from the dataset prior to subsequent spatial filtering. After excluding the sites beyond the known distribution range of the tufted deer in China as described in Classification and Distribution of Chinese Mammals (Wei 2022), a total of 452 occurrence points for the tufted deer were initially collected. To mitigate the effects of spatial autocorrelation due to closely spaced points (Yang, Luo, et al. 2025), Species Distribution Modeling Tools (SDMtools, v. 2.5) in ArcGIS (v. 10.8) software were employed to exclude occurrence points with inter‐point distances of less than 1 km (Song et al. 2025). After thinning, 429 occurrence points were retained (Figure 1; Table S1) and stored in CSV format.

### 2.2 Collection and Processing of Environmental Variables

Based on the biological characteristics of the tufted deer, 28 environmental variables were initially selected (Table 1), which included bioclimatic, geographical, hydrological, vegetational and anthropogenic factors. The bioclimatic variable data (Bio1~Bio19) were downloaded from the WorldClim climate database (http://www.Worldclim.org), with a spatial resolution of 2.5 min and approximately 4.5 km^2^. The geographical data included aspect, slope and altitude. The DEM layers at a resolution of 30 m were sourced from the Geospatial Data Cloud platform of the Computer Network Information Center, Chinese Academy of Sciences (https://www.gscloud.cn), and the slope and aspect were calculated from the DEM layers using ArcGIS software. The hydrological source (Dis_water) layer was downloaded from the National Geomatics Center of China (https://www.ngcc.cn), and the Dis_water was calculated as the Euclidean distance to water derived from the water source layers in ArcGIS software. The vegetation data included the vegetation fractional cover (VFC), which was derived from the Resources and Environmental Science Data Center (https://www.resdc.cn). The anthropogenic data included the human footprint (HFP), Gross Domestic Product (GDP), Population Density (PD), and Distance to Road Source (Dis_road). The HFP layer (~1 km) was obtained from the NASA Socio‐economic Data and Applications Center (https://www.earthdata.nasa.gov/centers/sedac‐daac). The GDP layer (~1 km) was obtained from the National Earth System Science Data Center (http://www.geodata.cn). The PD layer (~1 km) was obtained from Worldpop (Worldpop, https://www.worldpop.org/). The road layers were obtained from the National Geomatics Center of China (https://www.ngcc.cn), and the Dis_road data were calculated as the Euclidean distance to roads derived from the road layers in ArcGIS software.

**TABLE 1:** Environment variables for the Maxent modeling.

Environmental factor	Description	Unit
Bio1	Annual Mean Temperature	°C
Bio2	Mean Diurnal Range (Mean of monthly (max temp—min temp))	°C
Bio3	Isothermality (Bio2/Bio7) (* 100)	°C
Bio4	Temperature Seasonality (Standard Deviation * 100)	°C
Bio5	Max Temperature of Warmest Month	°C
Bio6	Min Temperature of Coldest Month	°C
Bio7	Temperature Annual Range (BIO5 – BIO6)	—
Bio8	Mean Temperature of Wettest Quarter	°C
Bio9	Mean Temperature of Driest Quarter	°C
Bio10	Mean Temperature of Warmest Quarter	°C
Bio11	Mean Temperature of Coldest Quarter	°C
Bio12	Annual Precipitation	mm
Bio13	Precipitation of Wettest Month	mm
Bio14	Precipitation of Driest Month	mm
Bio15	Precipitation Seasonality (Coefficient of Variation)	mm
Bio16	Precipitation of Wettest Quarter	mm
Bio17	Precipitation of Driest Quarter	mm
Bio18	Precipitation of Warmest Quarter	—
Bio19	Precipitation of Coldest Quarter	—
Altitude	Altitude	m
Slope	Gradient of the terrain	°
Aspect	Direction the slope faces	—
PD	Population Density	Person/km^2^
GDP	Gross domestic product	million RMB
HFP	Human footprint index	dimensionless index
VFC	Vegetation fractional cover	%
Dis_water	The Euclidean distance to roads	km
Dis_road	The Euclidean distance to water	km

*Note:* The grayed parts are the finally selected environmental factors.

### 2.3 Collinearity Analysis of Environmental Factors

**FIGURE 2:**
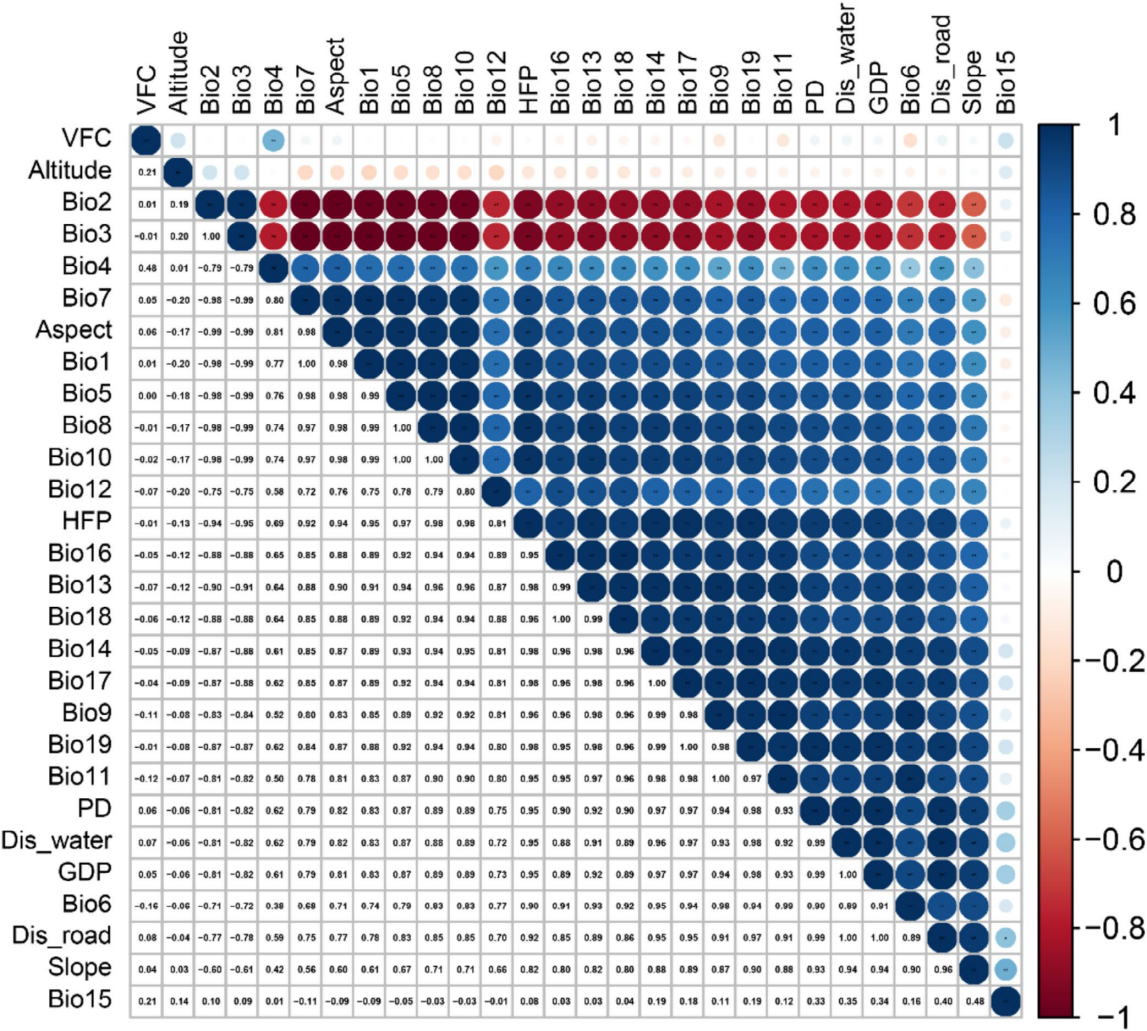
Correlation matrix of the environmental variables (The positive correlations are displayed in blue color and the negative correlations in a red color. The color intensity and the sizes of the circles are proportional to the correlation coefficients).

The coordinate system was unified (GCS_WGS_1984) and the spatial resolution was resampled (2.5 min, ~4.5 km^2^) in ArcGIS software and the environmental variable layers were clipped according to the range of China. The raster data were uniformly converted into ASCII format as required by Maxent (v. 3.4.4). To reduce multicollinearity and increase the robustness of model‐predicted relative probability of occurrence outputs, the environmental variables with high correlations but low model contribution scores were removed before the model analyses (Harisena et al. 2021). A correlation analysis was performed using ENMTools (https://github.com/danlwarren/ENMTools, v. 1.4), and the correlation coefficients were calculated (Figure 2) (West et al. 2016). The contribution rate of each variable was assessed in Maxent by the jackknife method using the occurrence points and the 28 environmental variables (Yaqin et al. 2025). The variables with very high correlations (|*r*| ≥ 0.85) but low model scores (< 1%) were removed (Song et al. 2025). If two predictors were highly correlated, the one with the higher model contribution score was retained (Shi et al. 2023). A total of eleven environmental variables were finally selected to construct the final models, which included six bioclimatic, two geographical, one vegetation, and two human activity variables (Table 1).

### 2.4 Model Parameter Optimization and Accuracy Evaluation

The use of default parameters can lead to overfitting and high omission rates when implementing MaxEnt models (Tao et al. 2024). The regularization multiplier (RM) and feature combination (FC) parameters in MaxEnt were used to optimize the analysis of the model (Song et al. 2025). There are the following five feature types in MaxEnt models: linear (L), quadratic (Q), hinge (H), product (P), and threshold (T). We set the RM to be between 0.5 and 4, increasing it by increments of 0.5 each time (Song et al. 2025). The feature combinations of L, LQ, H, LQH, LQHP, and LQHPT were selected. Combined with the 8 values of RM, this resulted in a total of 48 parameter combinations. The 48 parameter combinations were input into ENMeval (v. 2.0.4) for comprehensive testing. We used the delta from the Akaike information criterion (AIC), AICc, and the difference between training and testing AUC (diff_AUC) to evaluate the model's fitting degree and complexity for model‐predicted relative probability of occurrence (Lai et al. 2022; Shi et al. 2023). We imported the distribution data of tufted deer and the 11 environmental variables into MaxEnt and set the parameters to the optimal combination (ΔAICc = 0). This study allocated 75% of the occurrence points for the MaxEnt model‐predicted relative probability of occurrence for the tufted deer, while the remaining 25% was reserved as a test set to validate predictive performance (Jiang et al. 2025). A total of 10,000 points were randomly selected as background points, and bootstrap was selected as the replicated run type, with 10 repetitions (Song et al. 2025). The final simulation result was the mean of 10 repetitions.

The quality of the model results was evaluated by the area under the ROC curve (AUC). The AUC value is independent and is not affected by the critical value in the model and can be used to evaluate between model predictions and occurrence records (Zhu et al. 2017; Wan, Wang, et al. 2021). The range of the AUC is between 0 and 1 (Shi et al. 2023). An AUC < 0.7 suggests that the prediction performance is extremely poor; values between 0.7 and 0.8 indicate moderate performance; values between 0.8 and 0.9 suggest good performance; and values between 0.9 and 1.0 indicate excellent performance (Shi et al. 2023).

### 2.5 Analysis of Model‐Predicted Relative Probability of Occurrence and GAP for Tufted Deer in China

The results of the MaxEnt modeling for the tufted deer's model‐predicted relative probability of occurrence in China were formatted as ASCII (.asc) files and imported into ArcGIS software. Simultaneously, the provincial boundary layer of China (GS 20240650) was imported to generate a map of model‐predicted relative probability of occurrence for tufted deer in China. The results, ranging from 0 to 1, indicated higher species presence probabilities with larger values. The model‐predicted relative probability of occurrence was reclassified and divided into the following four levels by the natural breaks (Jenks) method: high (0.5–1), moderate (0.25–0.5), low (0.1–0.25), and very low (0–0.1) model‐predicted relative probability of occurrence zones (Zhang et al. 2022; Song et al. 2025). The zones and proportion of model‐predicted relative probability of occurrence zones for each level were calculated, and a distribution map was produced. This study defined the top eight provincial administrative regions with moderate‐to‐high model‐predicted relative probability of occurrence zones (MHZ) for tufted deer as priority zones for conservation. Furthermore, the MaxEnt model provided the response curves between the probability of species presence and the dominant environmental variables (Wang et al. 2024). Areas with predicted probability > 0.5 were classified as high model‐predicted relative probability of occurrence zones in the model output, and the corresponding environmental variable range was treated as a priority range for further assessment rather than a direct measure of habitat quality (Wang et al. 2024).

GAP analysis allows for a more general assessment of conservation effectiveness (Rodrigues, Akçakaya, et al. 2004; Wang et al. 2024). GAP analysis identifies the gap between the protection of high model‐predicted relative probability of occurrence zones by nature reserves and established conservation targets, which provides a measure of conservation effectiveness. Areas of model‐predicted priority not overlapping protected zones that are not covered by nature reserves are called unprotected priority areas. For species, if the percentage of a species' model‐predicted range covered by nature reserves meets the conservation target, the conservation effect is considered good; conversely, it indicates that there is a conservation vacancy for the species (Rodrigues, Andelman, et al. 2004; Wang et al. 2024). A total of 1028 nature reserves and 8 national parks were involved in this study, and the data were mainly obtained from Resource and Environmental Science Data Platform (http://www.resdc.cn). In order to show the conservation status of tufted deer in China, the map of model‐predicted relative probability of occurrence for the tufted deer was overlaid with the vectorial boundaries of China's wilderness areas in ArcGIS software, resulting in a map of conservation vacancies for GAP analysis, which provides a theoretical reference basis for the development of scientific conservation and management of tufted deer (Liang and Li 2024; Wang et al. 2024).

## 3 Results

### 3.1 The Distribution Sites of the Tufted Deer in China

Based on data from the CRLB, NSII, and GBIF, a total of 429 occurrence points for the tufted deer were documented across 16 provinces, autonomous regions, or municipalities in China. The distribution data indicated that Sichuan had the highest number of occurrence points (*n* = 159), followed by Yunnan (*n* = 43), Gansu (*n* = 30), Zhejiang (*n* = 29), and Guangxi (*n* = 28) (Table S1). This information serves as a basis for constructing MaxEnt models for the species.

### 3.2 Model Optimization and Accuracy Evaluation

In this study, based on 429 distribution records and 11 environmental variables, the MaxEnt model, optimized with the ENMeval package, was used to predict the model‐predicted relative probability of occurrence of tufted deer. Among the 48 candidate models, all exhibited statistical significance. The default model setting for tufted deer was M_Default (regularization multiplier = 1, feature class combination = L, Q, P, and H) with a mean AUC value of 0.941 ± 0.049 and a testing AUC value of 0.892 ± 0.049. When running the ENMeval package in R, only one model met the AICc criteria (ΔAICc = 0). Therefore, the selected MaxEnt model setting for *tufted deer* was M_Optimized (regularization multiplier = 1.5, feature class combination = L, Q, P, T, and H). The mean AUC value was 0.948 ± 0.050, and the testing AUC value was 0.898 ± 0.050 (Figure S1). These results indicate that the optimized MaxEnt model provides a high level of predictive agreement between the model outputs and the known occurrence records of the species, demonstrating strong performance under the chosen parameterization (Table S2).

### 3.3 Environmental Variable Selection and Analysis of Environmental Factors Associated With Tufted Deer Distribution

**FIGURE 3:**
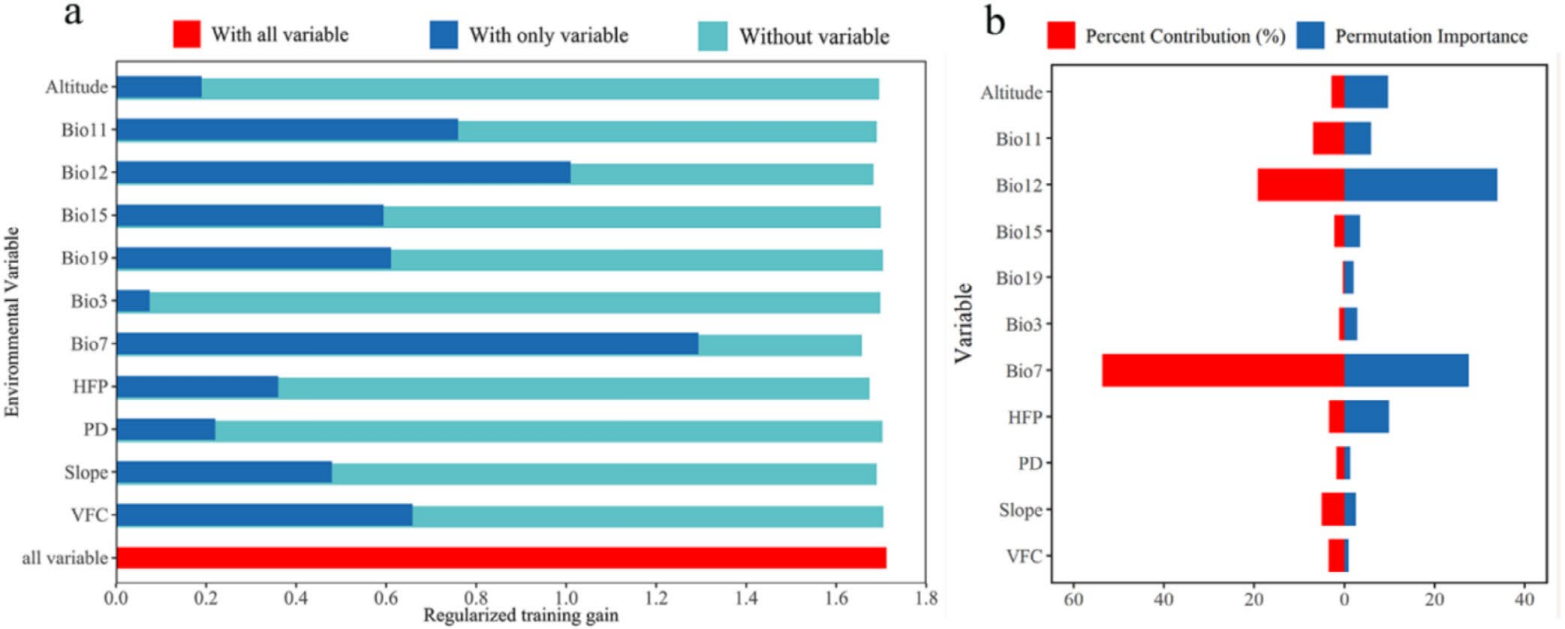
Jackknife of test gain for the environmental variables (a) and the relative contribution of environmental factors in constructing the MaxEnt model for tufted deer in China (b).

The jackknife method in the MaxEnt model was used to evaluate the relative importance of 11 environmental factors in relation to the distribution records of tufted deer in the current period (Figure 3). The model also calculated the Percent Contribution (PC) and Permutation Importance (PI) for each environmental variable (Table S3). The jackknife test indicated that Bio7, Bio12, Bio11, VFC, Bio19, and Bio15 were the most influential variables in terms of model performance (Figure 3a). The six variables with the highest PC were Bio7 (53.6%), Bio12 (19.2%), Bio11 (6.9%), Slope (5.0%), VFC (3.5%), and HFP (3.4%), with a cumulative contribution of 91.6% (Figure 3b, Table S3). A comprehensive evaluation showed that the leading environmental factors associated with higher model‐predicted relative probability of occurrence of tufted deer were Bio7, Bio12, Bio11, Slope, VFC, and HFP.

**FIGURE 4:**
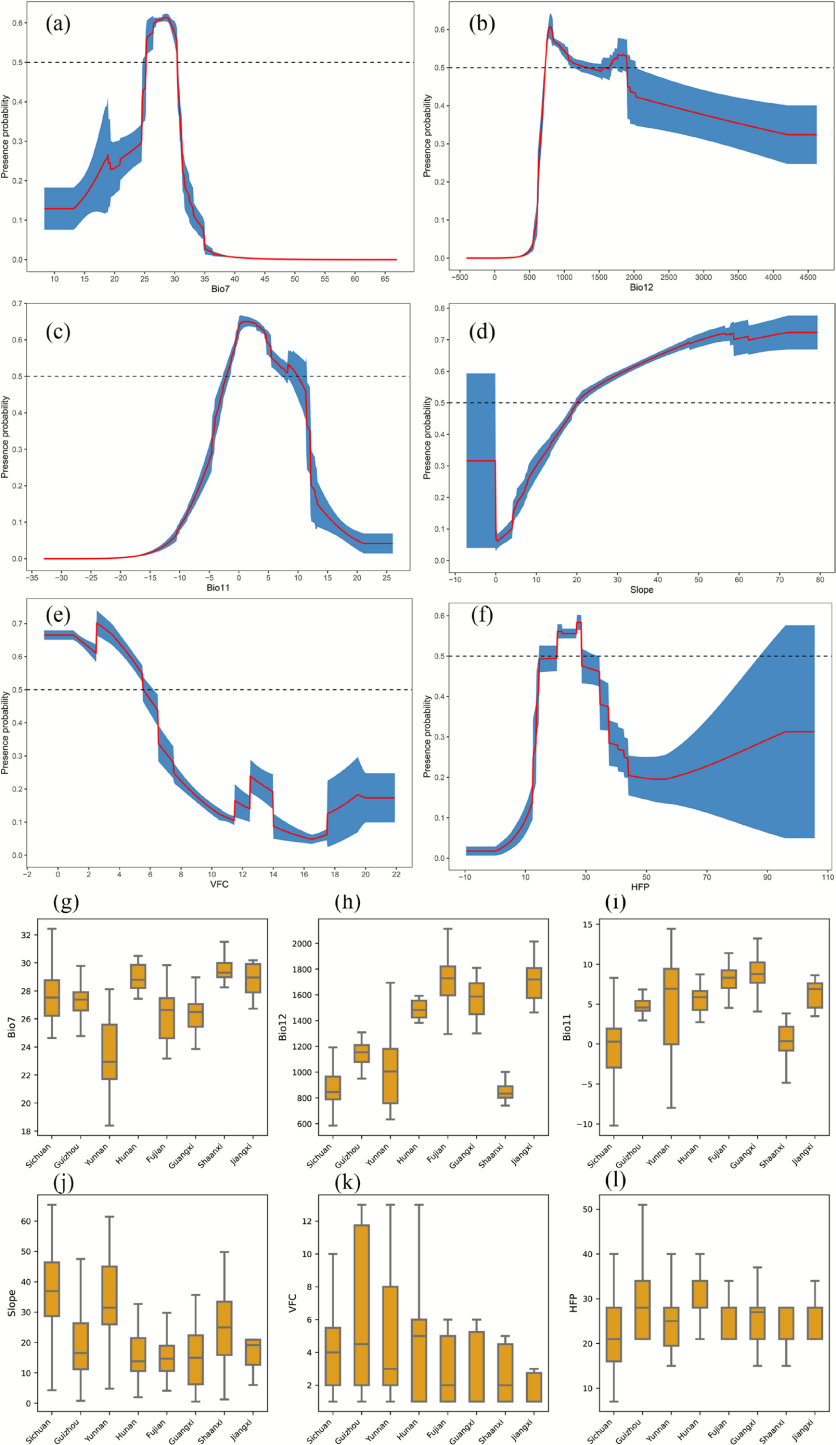
The response curves of the major climate factors including Bio7 (a), Bio12 (b), Bio11 (c), Slope (d), VFCI (e), and HFP (f) (red is the response curve and blue is the standard error); Comparison and analysis of the environmental factors across different provinces including Bio7 (g), Bio12 (h), Bio11 (i), Slope (j), VFC (k), and HFP(l).

The response curves derived from the MaxEnt model revealed that tufted deer exhibited the highest model‐predicted relative probability of occurrence in areas with temperature annual range (Bio7) of 25.27–30.42 (Figure 4a). The association with annual precipitation (Bio12) was complex, with elevated predicted occurrence probabilities in two ranges: 725.64–1324.26 mm and 1651.23–1897.72 mm (Figure 4b). The response to the mean temperature of the coldest quarter (Bio11) was parabolic, with elevated predicted probabilities between −2.04°C and 9.94°C (Figure 4c). The slope followed a semi‐parabolic trend, with predicted occurrence probability increasing with slope and peaking between 19.96° and 74.18° (Figure 4d). Similarly, vegetation fractional cover (VFC) displayed a semi‐parabolic trend, with elevated model‐predicted relative probability of occurrence at VFC values between 0.00% to 5.53% (Figure 4e). The response to the Human Footprint Index (HFP) was parabolic, with higher predicted occurrence probability in the range of 20.47–28.53 (Figure 4f).

Furthermore, we selected eight provinces with large areas of model‐predicted moderate and high occurrence probability zones (MHZ) for the tufted deer for a comparative analysis of environmental factors (Figure 4g–l; Table 2). As illustrated in Figure 4g, there were notable differences in the annual temperature range across the various provinces. Shaanxi exhibited the highest annual temperature range, while Yunnan showed the lowest. Tufted deer in Sichuan experienced relatively lower annual precipitation (Bio12) and Mean Temperature of the Coldest Quarter (Bio11), whereas tufted deer in Fujian and Guangxi provinces had comparatively higher values for these variables (Figure 4h–i). Sichuan exhibited the highest mean slope, while Hunan showed the lowest (Figure 4j). Hunan exhibited the highest VFC value, while Shaanxi showed the lowest (Figure 4k). Guizhou exhibited the highest HFP index, while Sichuan showed the lowest (Figure 4l).

**TABLE 2:** Suitability of model‐predicted relative probability of occurrence zones for tufted deer in different provinces of China.

	Province	HPZ	MPZ	LPZ	MHZ	SUM
Area (10^4^ km^2^)	Sichuan	12.34	9.52	16.29	21.86	38.15
	Guizhou	4.65	9.25	2.08	13.90	15.98
	Yunnan	3.73	8.29	14.99	12.02	27.01
	Hunan	3.46	6.06	6.79	9.53	16.32
	Fujian	3.13	4.61	1.73	7.74	9.47
	Guangxi	2.23	4.98	5.34	7.21	12.55
	Shannxi	4.67	2.35	2.00	7.01	9.02
	Jiangxi	1.77	4.73	5.47	6.49	11.96
Proportion (%)	Sichuan	25.38	19.59	33.52	44.98	78.50
	Guizhou	26.41	52.55	11.79	78.96	90.75
	Yunnan	9.48	21.03	38.03	30.50	68.53
	Hunan	16.35	28.63	32.07	44.98	77.05
	Fujian	25.80	37.97	14.21	63.77	77.98
	Guangxi	9.37	20.96	22.49	30.33	52.82
	Shannxi	29.86	15.02	12.81	44.87	57.68
	Jiangxi	10.58	28.34	32.76	38.91	71.67

Abbreviations: HPZ, High model‐predicted relative probability of occurrence zones (0.5–1.0); LPZ, Low model‐predicted relative probability of occurrence zones (0.1–0.25); MHZ, Combined moderate and high model‐predicted relative probability of occurrence zones (HPZ + MPZ); MPZ, Moderate model‐predicted relative probability of occurrence zones (0.25–0.5); SUM, Total model‐predicted relative probability of occurrence zones above 0.1 threshold (HPZ + MPZ + LPZ).

### 3.4 Analysis of Model‐Predicted Distribution for Tufted Deer in China and Major Provinces

**FIGURE 5:**
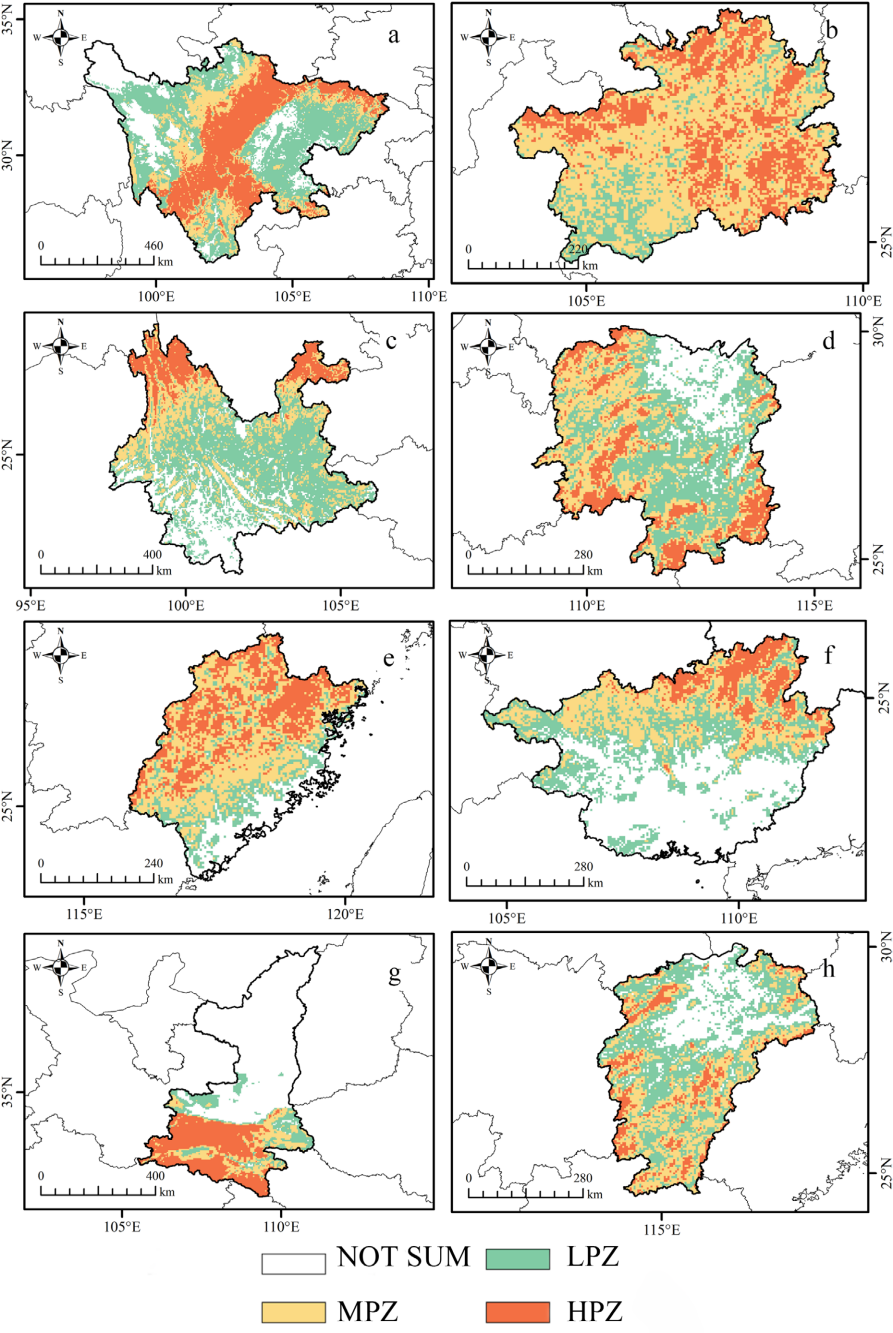
Top 8 provinces with medium and high model‐predicted relative probability of occurrence zones for Tufted Deer (a, Sichuan; b, Guizhou; c, Yunnan; d, Hunan; e, Fujian; f, Guangxi; g, Shannxi; h, Jiangxi). HPZ, High model‐predicted relative probability of occurrence zones (0.5–1.0); LPZ, Low model‐predicted relative probability of occurrence zones (0.1–0.25); MPZ, Moderate model‐predicted relative probability of occurrence zones (0.25–0.5); NOT SUM, Total model‐predicted relative probability of occurrence zones under 0.1 threshold.

The areas with higher model‐predicted relative probability of occurrence for the tufted deer are primarily located in southwest, south‐central, and southeast China. Within this range, moderate‐to‐high model‐predicted relative probability of occurrence zones (MHZ) are concentrated in the central and southern regions (Figure S2). An analysis of key provinces revealed that Sichuan contained the largest area of MHZ (21.86 × 10^4^ km^2^), followed by Guizhou (13.90 × 10^4^ km^2^), Yunnan (12.02 × 10^4^ km^2^), and Hunan (9.53 × 10^4^ km^2^). Provinces with smaller, yet significant, MHZ areas included Fujian (7.74 × 10^4^ km^2^), Guangxi (7.21 × 10^4^ km^2^), Shaanxi (7.01 × 10^4^ km^2^), and Jiangxi (6.49 × 10^4^ km^2^) (Figure 5; Table 2).

The core of the species' model‐predicted higher occurrence probability zones lies in the mountainous southwestern provinces. In Sichuan, high model‐predicted relative probability of occurrence zones align with the western, southern, and southeastern mountain ranges, while moderate model‐predicted relative probability of occurrence zones occur in the hills and transitional areas around the Sichuan Basin. Very low model‐predicted relative probability of occurrence zones are largely confined to the basin's core and high‐altitude plateaus. In total, areas with model‐predicted relative probability of occurrence zones above 0.1 covered 78.50% (38.15 × 10^4^ km^2^) of the province, comprising high model‐predicted relative probability of occurrence zones (25.38%), moderate model‐predicted relative probability of occurrence zones (19.59%), and low model‐predicted relative probability of occurrence zones (33.52%). In neighboring Guizhou, 90.75% of the province was classified as model‐predicted relative probability of occurrence above 0.1 (15.98 × 10^4^ km^2^), with high model‐predicted relative probability of occurrence zones concentrated in the central‐northern, northeastern, and central‐southern regions. In Yunnan, areas with model‐predicted relative probability of occurrence above 0.1 covered 68.53% of the province (27.01 × 10^4^ km^2^), with high model‐predicted relative probability of occurrence zones concentrated in the northeastern and central mountains, as well as the fringes of the Hengduan Mountains.

Eastward from this core, model‐predicted relative probability of occurrence zones extend across several provinces. In Hunan, high model‐predicted relative probability of occurrence zones were scattered throughout the southwestern mountains, with low model‐predicted relative probability of occurrence zones covering the central and northern plains; overall, areas with a model‐predicted relative probability of occurrence above 0.1 covered 77.05% of the province (16.32 × 10^4^ km^2^). In Fujian, the spatial pattern of predicted occurrence probability followed a similar east–west gradient, with high model‐predicted relative probability of occurrence zones in the western and northern mountains and low model‐predicted relative probability of occurrence zones in the coastal plains; 77.98% of the province was within the > 0.1 predicted probability threshold (9.47 × 10^4^ km^2^). In Guangxi, higher model‐predicted relative probability of occurrence zones were concentrated in mountainous and hilly regions, covering 52.82% of the territory (12.55 × 10^4^ km^2^). In Shaanxi, representing the northern extent, high model‐predicted relative probability of occurrence zones were predominantly located in the southern Qinba region, with low model‐predicted relative probability of occurrence zones in the Guanzhong Plain and Loess Plateau. In total, 57.68% of Shaanxi had a model‐predicted relative probability of occurrence above 0.1 (11.96 × 10^4^ km^2^); this comprised high, moderate, and low model‐predicted relative probability of occurrence zones, which accounted for 44.87%, 29.86%, and 15.02% of the total provincial area, respectively. In Jiangxi, high model‐predicted relative probability of occurrence zones occurred in the northwestern, southern, and eastern (Luoxiao) mountains, while low model‐predicted relative probability of occurrence zones corresponded to highly urbanized regions; 71.67% of the province's area had a model‐predicted relative probability of occurrence above 0.1 (11.96 × 10^4^ km^2^).

### 3.5 Analysis of Conservation Gaps for Tufted Deer in China and Major Provinces

**FIGURE 6:**
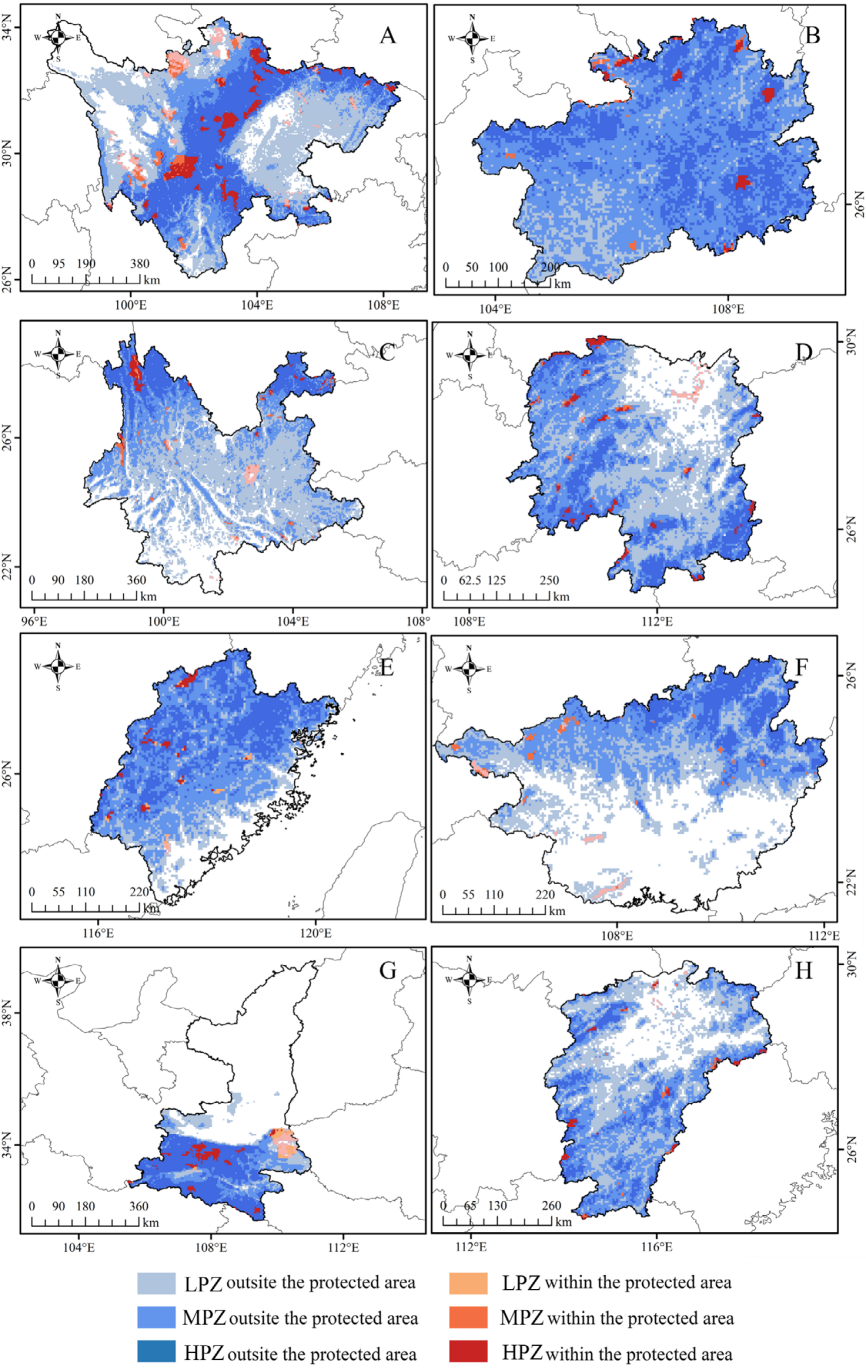
Protection gaps in the top eight provinces with moderate and high model‐predicted relative probability of occurrence zones for Tufted Deer (A, Sichuan; B, Guizhou; C: Yunnan; D, Hunan; E, Fujian; F, Guangxi; G, Shannxi; H, Jiangxi); (HPZ_P: High model‐predicted relative probability of occurrence zones within protected areas (0.5–1.0); MPZ_P: Moderate model‐predicted relative probability of occurrence zones within protected areas (0.25–0.5); LPZ_P: Low model‐predicted relative probability of occurrence zones within protected areas (0.1–0.25)).

At the national scale, our gap analysis reveals that the vast majority of zones with higher model‐predicted relative probability of occurrence for the tufted deer (93.98%) lies outside of existing nature reserves. This conservation gap is particularly pronounced for MHZ, 93.54% of which falls outside current protected areas (Figure S3). This pattern of limited coverage is also evident at the provincial level. Sichuan, which hosts the most MHZ, has the largest extent of model‐predicted higher‐probability areas within reserve boundaries. Protection ratios are even lower for other key provinces, including Shaanxi (5.24%), Yunnan (4.20%), Hunan (3.88%), and Fujian (2.58%). Guizhou has the lowest proportion of protected MHZ at just 2.01%. In terms of absolute area, Sichuan contains the largest portion of MHZ overlapping protected zones (3.49 × 10^4^ km^2^), followed by Yunnan (0.93 × 10^4^ km^2^) and Shaanxi (0.91 × 10^4^ km^2^). Considerably smaller protected MHZ are found in Hunan (0.53 × 10^4^ km^2^), Guizhou (0.30 × 10^4^ km^2^), and Guangxi (0.30 × 10^4^ km^2^). The smallest extents of MHZ overlapping protected zones are in Fujian and Jiangxi (0.22 × 10^4^ km^2^ each) (Figure 6; Table 3).

**TABLE 3:** Proportion and area of model‐predicted occurrence probability zones for tufted deer within protected areas in China.

	Province	HPZ_P	MPZ_P	LPZ_P	MHZ_P	SUM_P
Area (10^4^ km^2^)	Sichuan	1.66	0.71	1.12	2.37	3.49
	Guizhou	0.18	0.10	0.02	0.28	0.30
	Yunnan	0.33	0.30	0.30	0.63	0.93
	Hunan	0.30	0.10	0.13	0.40	0.53
	Fujian	0.13	0.07	0.02	0.20	0.22
	Guangxi	0.18	0.10	0.02	0.28	0.30
	Shaanxi	0.43	0.20	0.28	0.63	0.91
	Jiangxi	0.13	0.06	0.03	0.19	0.22
Proportion_P (%)	Sichuan	13.45	7.46	6.88	10.84	9.15
	Guizhou	3.87	1.08	0.96	2.01	1.88
	Yunnan	8.85	3.62	2.00	5.24	3.44
	Hunan	8.67	1.65	1.91	4.20	3.25
	Fujian	4.15	1.52	1.16	2.58	2.32
	Guangxi	8.07	2.01	0.37	3.88	2.39
	Shaanxi	9.21	8.51	14.00	8.99	10.09
	Jiangxi	7.34	1.27	0.55	2.93	1.84

Abbreviations: HPZ_P, High model‐predicted relative probability of occurrence zones within protected areas (0.5–1.0); LPZ_P, Low model‐predicted relative probability of occurrence zones within protected areas (0.1–0.25); MHZ_P, Combined moderate and high model‐predicted relative probability of occurrence zones within protected areas (HPZ + MPZ); MPZ_P, Moderate model‐predicted relative probability of occurrence zones within protected areas (0.25–0.5); SUM_P, Total model‐predicted relative probability of occurrence zones above 0.1 threshold within protected areas (HPZ + MPZ + LPZ).

## 4 Discussion

### 4.1 Environmental Factors Associations With Model‐Predicted Relative Probability of Occurrence for the Tufted Deer in Different Provinces

In this study, the MaxEnt model was used to identify key environmental variables associated with the model‐predicted relative probability of occurrence of the tufted deer. The strongest predictors by percent contribution included annual temperature range (Bio7), annual precipitation (Bio12), mean temperature of the coldest quarter (Bio11), slope, vegetation fractional cover (VFC), and the human footprint index (HFP), among which Bio7 had the highest percent contribution (53.6%). Previous studies have reported that within the Gongga Mountain National Nature Reserve in Sichuan Province, China, strong statistical associations were reported between occurrence records and precipitation seasonality, vegetation types, NDVI, mean temperature of the driest quarter, total annual precipitation, and isothermality; collectively, hydrothermal conditions and vegetation structure were associated with higher predicted occurrence probabilities (You et al. 2022). In addition, other studies have shown that in Hunan Province, occurrence patterns were associated with elevation (Alt), mean diurnal temperature range (Bio2), vegetation types (Veg), coefficient of variation of precipitation (Bio15), isothermality (Bio3), and mean temperature of the driest quarter (Bio9) (Yang, Deng, et al. 2025). In the present study, elevated model‐predicted relative probability of occurrence was observed at annual precipitation ranges of 725–1324 mm and 1651–1897 mm, and at mean temperature of the coldest quarter ranged from −2.04°C to 9.94°C, which is consistent with previously reported hydrothermal associations.

Comparative analyses across different regions of China further revealed that the heterogeneity of key environmental variables among provinces corresponded with notable differences in the model‐predicted distribution patterns of the tufted deer. In Shaanxi Province, areas with higher predicted occurrence probabilities exhibited the highest annual temperature range, which may suggest a broader tolerance to thermal fluctuations. In contrast, Sichuan Province was characterized by lower annual precipitation and lower mean temperature of the coldest quarter, conditions typically found in cool and humid mountainous environments. Previous studies reported the presence of tufted deer in the Changqing National Nature Reserve in Shaanxi Province, where activity rhythm studies showed bimodal peaks at 08:00–09:00 and 18:00–19:00, a crepuscular pattern possibly related to temperature variation through the day (Lu et al. 2023). Similarly, in Hunan Province, the diurnal activity intensity of tufted deer showed significant variation, with the lowest relative abundance index recorded at 04:00–06:00 and the highest at 18:00–20:00 (Xiang et al. 2024). These findings, together with the present model results, suggest a tendency toward cooler conditions. Furthermore, previous research demonstrated that tufted deer favored habitats with high vegetation cover, and increasing vegetation cover significantly improved habitat suitability (Xiang et al. 2022). In agreement with these patterns, the present study found vegetation fractional cover to be highest in Hunan and lowest in Shaanxi, with extensive hilly and forested landscapes in southern China overlapping substantially with model‐predicted relative probability of occurrence zones. This appears consistent with the species' use of areas offering substantial understory and diverse food resources.

### 4.2 Distribution Pattern of Model‐Predicted Occurrence Probability Zones for Tufted Deer in Different Provinces of China

Based on the MaxEnt model, this study systematically evaluated the predicted zones with model‐predicted relative probability of occurrence for the tufted deer in China. The results showed that its zones with moderate‐to‐high model‐predicted relative probability of occurrence were primarily concentrated in the mountainous and hilly regions of southwestern, southern, and southeastern China, particularly in the central and southern areas of Sichuan, Guizhou, Yunnan, and Hunan provinces. These regions are generally characterized by pronounced topographic variation and complex terrain. Previous studies reported the detection of tufted deer and other vertebrate species by analyzing environmental DNA from the gut contents of leeches collected in the Ailao Mountains Nature Reserve in Yunnan Province, confirming its presence in Yunnan. Ji et al. (2022) reported that tufted deer were more frequently recorded in areas located farther from the edges of protected areas and at higher elevations. In addition, long‐term monitoring using 9 years of infrared camera data from Gongga Mountain National Nature Reserve in Sichuan Province provided clear evidence of the stable presence of tufted deer, with an estimated area of 1038.40 km^2^ and over 10,000 photographic records. These findings documented the species' extensive activity within the alpine forest ecosystems of the eastern Qinghai‐Tibet Plateau (You et al. 2022). Furthermore, the MaxEnt model was used to map predicted distributions for key prey species in the process of rewilding the South China tiger ( *Panthera tigris amoyensis* ), including the tufted deer. The results indicated that tufted deer had distinct areas of higher predicted occurrence in China and that the species represents an important component of the prey community within potential rewilding sites for South China tigers. This indicated that tufted deer had stable or potential distributions in the middle and lower reaches of the Yangtze River and adjacent regions, fulfilling essential ecological functions as a prey base for apex predators such as the South China tiger (Luo et al. 2024). Collectively, these findings, together with the present study, suggested that tufted deer are often associated with mountainous ecosystems characterized by complex topography, mid‐to‐high elevations, good forest cover, and low levels of human disturbance.

From a spatial perspective, model results indicated over 90% of Guizhou Province was predicted to offer environmental conditions potentially favorable for the tufted deer, indicating that the low mountain and hilly environments formed by its karst landforms may support high ecological carrying capacity for the species. Previous research based on infrared camera monitoring conducted between December 2018 and April 2021 in Leigongshan National Nature Reserve clearly demonstrated a stable distribution of tufted deer in this area. The species exhibited a relatively high relative abundance index, was one of the commonly recorded species in the reserve, and was detected across high (1600–2080 m), mid (1200–1600 m), and low elevations (790–1200 m), reflecting a broad vertical distribution range, strong ecological adaptability, and a certain population size (Li et al. 2022). Similarly, infrared camera surveys conducted in 2019 in Fanjingshan and Chishui Alsophila National Nature Reserves showed that tufted deer were widely distributed and relatively stable in population across both sites. The occupancy rate reached 0.58, indicating that tufted deer were among the dominant medium‐to‐large mammal species in the region and exhibited high habitat adaptability and frequent activity (Wan, Li, et al. 2021). In addition, camera trap monitoring and activity rhythm analysis carried out in Xishui National Nature Reserve recorded tufted deer as one of the main medium‐sized mammals among 28 detected mammal species. Their activity was mainly concentrated in the evening, demonstrating a typical crepuscular activity pattern and stable presence in the reserve (Mu et al. 2019). Together with the present study, these findings collectively indicated that tufted deer maintain a widespread distribution pattern across Guizhou Province. This underscores the province's importance in the conservation and management of the species and provides a solid foundation for the development of science‐based conservation strategies and ecological restoration efforts.

### 4.3 Conservation Gaps of Tufted Deer in China

This study, through a nationwide gap analysis, found that 93.98% of the areas with high model‐predicted relative probability of occurrence for the tufted deer were located outside of existing nature reserves. The protection gap was particularly pronounced for MHZ, 93.54% of which had not yet been incorporated into the current protected area network. These findings suggest a substantial shortfall in China's existing nature reserve system in covering key areas likely important for the species, highlighting the urgent need to optimize the spatial layout of protected areas and improve habitat representativeness and connectivity. At the provincial scale, although Sichuan Province contained the largest extent of MHZ for the tufted deer, only 10.84% of these zones were under protection. In other key provinces such as Guizhou, Fujian, and Hunan, the proportion of protected MHZ was even lower—less than 5%, indicating that the majority of areas with relatively high model‐predicted relative probability of occurrence remained outside the current protected area network. This spatial mismatch was representative of patterns observed in other species with high ecological niche suitability and underscored the limitations of traditional protected area designation strategies that rely primarily on species occurrence points, particularly in addressing future conservation needs.

Previous studies had conducted conservation gap assessments for key protected species in various local regions. Based on the distribution ranges of 19,039 terrestrial vertebrate species, it was found that approximately 75% of species' distributions in global border regions were not covered by nature reserves. This gap was particularly prominent in tropical regions of Southeast Asia and West Africa, indicating substantial biodiversity conservation gaps in border areas worldwide (Li et al. 2025). In addition, under the context of climate change, an evaluation of the distribution patterns of threatened ungulates in China using multidimensional biodiversity indices at a 0.1° resolution showed that less than 30% of the highest‐priority conservation areas were covered by existing nature reserves. In eastern China, only 21.5%–22.2% of these high‐priority areas were protected, highlighting the existence of persistent conservation gaps under intense human disturbance and the urgent need to enhance protection in critical areas (Zhang et al. 2025). Another study assessed the predicted suitability and conservation effectiveness of nature reserves for the endangered forest musk deer in western China using ecological niche models and ArcGIS techniques. The results showed pronounced variation in the predicted suitability within national nature reserves across western China, with Sichuan's reserves exhibiting the highest protection efficiency for highly suitable habitats, while substantial conservation gaps were found across all four provinces studied (Jiang et al. 2025). Furthermore, based on large‐scale infrared camera monitoring data from the Gaoligong Mountains, a multi‐species occupancy model and β‐diversity indices were used to evaluate the effects of human disturbance and environmental variables on the distribution of medium and large mammals. The results indicated that species richness increased during the dry season in low‐elevation areas (1700–2300 m), suggesting notable spatiotemporal conservation value in these zones. However, protection gaps remained in these key lowland zones, emphasizing the need for targeted conservation and habitat restoration efforts (Hu et al. 2025).

This study, together with the aforementioned research findings, suggests that there was a substantial gap in China's existing protected area network in covering areas with relatively high model‐predicted relative probability of occurrence for the tufted deer. This spatial mismatch is consistent with conservation gap patterns observed for similar species and regions in China and elsewhere. It indicates the urgent need to refine protected area planning, particularly under the increasing pressures from climate change and human disturbance, including the careful use of spatial prioritization and species distribution modeling to support decision‐making. In summary, this study identified a notable spatial gap in the current conservation system for the tufted deer and outlined candidate spatial priorities for its future conservation planning, which may inform ecological insights and valuable guidance for conservation policy.

### 4.4 Protection Countermeasures and Suggestions of Tufted Deer in China

Although the tufted deer has been listed as a National Class II Protected Species in China, this study indicates that the majority of zones with relatively high model‐predicted relative probability of occurrence remained outside the current network of nature reserves, suggesting a notable spatial gap in habitat protection. Therefore, there is an urgent need to refine the protected area system and enhance the coverage of priority conservation zones for this species. Specifically, it is recommended that newly identified moderate‐to‐high model‐predicted relative probability of occurrence zones—particularly in Sichuan, Guizhou, and Hunan provinces—be considered for the establishment of new nature reserves or expansion of existing boundaries to help address identified conservation gaps. To ensure the stability and adaptability of protected area functions, the concept of Systematic Conservation Planning (Villarreal‐Rosas et al. 2020) should be adopted, incorporating model‐based predictions, environmental heterogeneity, and human disturbance as key criteria for zoning and tiered management. In addition, a cross‐regional coordination mechanism should be established to facilitate information sharing and joint management among administrative regions. Furthermore, a refined habitat monitoring system should be developed, integrating infrared camera traps, satellite remote sensing, and unmanned aerial vehicles (UAVs) to monitor habitat quality and population dynamics in key areas in real time. This would enable timely evaluation of conservation effectiveness and inform adaptive management strategies, ultimately improving conservation efficiency and resilience under climate change.

The zones with model‐predicted relative probability of occurrence of the tufted deer were generally subject to varying degrees of human disturbance, with habitat fragmentation particularly evident in densely populated or rapidly developing regions. To alleviate the ecological pressure caused by human activities, the strategy of core protection and buffer zone management should be implemented in key distribution provinces (Fang et al. 2022). This approach involves restricting high‐intensity construction projects from entering core habitat areas while guiding the development of infrastructure such as roads and tourism facilities away from highly suitable zones. For already degraded or fragmented habitats, ecological restoration projects should be undertaken, including reforestation, restoration of native vegetation, and removal of ecological barriers, to enhance habitat connectivity and overall ecosystem functionality. At the policy level, the development of region‐specific ecological compensation mechanisms targeting small mountain‐dwelling ungulates such as the tufted deer should be promoted. These policies should encourage community participation in habitat conservation and restoration efforts, while also raising public awareness of ecological protection. Striking a balance between economic development and biodiversity conservation, it is essential to strengthen environmental education and foster community‐based co‐management initiatives. Promoting a model community co‐management and ecotourism would support both conservation goals and local development, contributing to the long‐term stability and sustainable management of tufted deer habitats.

## 5 Conclusion

This study systematically evaluated the zones with model‐predicted relative probability of occurrence and associated environmental factors of the tufted deer in China based on 429 occurrence records and 11 selected environmental variables using an optimized MaxEnt model. The model demonstrated excellent predictive performance, with an AUC value reaching 0.948, indicating high reliability of the results. The findings showed that the tufted deer's zones with moderate‐to‐high model‐predicted relative probability of occurrence were mainly concentrated in southwestern, south‐central, and southeastern China, with Sichuan, Guizhou, Yunnan, and Hunan provinces identified as the provinces with the largest extents of such zones. Model contribution analysis revealed that annual temperature range (Bio7), annual precipitation (Bio12), mean temperature of the coldest quarter (Bio11), slope, vegetation fractional cover (VFC), and human footprint index (HFP) cumulatively contributed 91.6% to the prediction. Among them, Bio7 has the highest single contribution (contribution rate of 53.6%). Elevated predicted occurrence probabilities were associated with an annual temperature range of 25.27–30.42, annual precipitation of 725–1324 mm and 1651–1897 mm, and mean temperature of the coldest quarter between −2.04°C and 9.94°C, conditions that in combination describe cooler and more humid environments. Analysis at the provincial scale revealed notable differences in the ranges of key environmental variables. For example, in Sichuan, predicted occurrence zones tended to coincide with lower temperatures and steeper slopes, while in Hunan, such zones aligned more with higher vegetation cover and areas showing higher human footprint index values. GAP analysis revealed that 93.98% of the zones with model‐predicted relative probability of occurrence nationwide are located outside current nature reserves, with MHZ protected at only 6.46% overall. Among major provinces, Sichuan had the highest MHZ protection rate at merely 10.84%, while Guizhou, Hunan, and Fujian had rates below 4%, indicating that current protection networks inadequately covered critical tufted deer habitats. Overall, this study provides a spatially explicit overview of the tufted deer's model‐predicted distribution patterns and associated environmental characteristics, alongside an assessment of conservation coverage. It underscored the urgent need to prioritize the expansion and optimization of protected area networks in high model‐predicted relative probability of occurrence zones, especially Sichuan, Guizhou, and Yunnan, and to promote spatial planning informed by species‐distribution‐model outputs to achieve more scientific, systematic, and efficient conservation management for this species.

## Author Contributions

**Yuangang Yang:** conceptualization (equal), data curation (equal), investigation (equal), methodology (equal), project administration (equal), supervision (equal), visualization (equal), writing – original draft (equal). **Peng Luo:** conceptualization (equal), data curation (equal), investigation (equal), methodology (equal), project administration (equal), visualization (equal), writing – original draft (equal). **Yu Zhao:** formal analysis (equal), investigation (equal), software (equal), validation (equal). **Hua Li:** formal analysis (equal), software (equal). **Yufang Yang:** software (equal), validation (equal). **Mengyao Li:** software (equal), validation (equal). **Tongzuo Zhang:** resources (equal). **Feng Jiang:** funding acquisition (equal), resources (equal), writing – review and editing (equal). **Zhangqiang You:** conceptualization (equal), data curation (equal), funding acquisition (equal), methodology (equal), supervision (equal), writing – review and editing (equal).

## Funding

This study was supported by the Scientific Research Initiation Project [QD2023A02], China Postdoctoral Science Foundation [2024T170992, 2023M743743], and the 2023 award fund of Qinghai Provincial Key Laboratory of Animal Ecological Genomics [QHEG‐2024‐05], the Mianyang Science and Technology Program (2023ZYDF076).

## Conflicts of Interest

The authors declare no conflicts of interest.

## Supporting information


**Table S1:** The occurrence record of the tufted deer in different provinces.
**Table S2:** Evaluation results of the MaxEnt model generated by ENMeval. AICc, Akaike The akaike information criterion corrected; AUC, The area under the subject curve; Dela.AlCc, AICc the minimum information criterion AICc value; diff.AUC, Average difference between the training and testing AUC; LQHP, Linear features + Quadratic features + Product features + Hinge features; LQPTH, Linear features + Quadratic features + Product features + Threshold features + Hinge features.
**Table S3:** Relative contribution of environmental variables to the final model.
**Figure S1:** ∆AICc values of the Maxent models under different regularization multipliers (RM) and feature combinations (FC); Receiver operating characteristic (ROC) curve; AUC curve (a, Default_AUC; b, ∆AICc values; c, Optimized_AUC).
**Figure 2**. Zones with Model‐Predicted Relative Probability of Occurrence for Tufted Deer in China. HPZ, High model‐predicted relative probability of occurrence zones (0.5–1.0); LPZ, Low model‐predicted relative probability of occurrence zones (0.1–0.25); MPZ, Moderate model‐predicted relative probability of occurrence zones (0.25–0.5); NOT SUM, Total model‐predicted relative probability of occurrence zones under 0.1 threshold.
**Figure S3:** Zones of vacancies in Predicted occurrence probability for Tufted Deer in China. (HPZ_P, High model‐predicted relative probability of occurrence zones within protected areas (0.5–1.0); LPZ_P, Low model‐predicted relative probability of occurrence zones within protected areas (0.1–0.25); MPZ_P, Moderate model‐predicted relative probability of occurrence zones within protected areas (0.25–0.5)).

**Data S1:** ece372850‐sup‐0002‐DataS1.csv.

## Data Availability

The data that supports the findings of this study are available in the Supporting Information of this article.
