# Supplementary material for: Distribution Pattern, Ecological Determinants and Conservation Gaps of Model‐Predicted Relative Probability of Occurrence Zones for the Tufted Deer (Elaphodus cephalophus) in China
Source: Ecol Evol. 2026 Jan 8;16(1):e72850. doi: 10.1002/ece3.72850 (PMC12782671; doi:10.1002/ece3.72850)
Supplement: Supplementary file 1 — Table S1: The occurrence record of the tufted deer in different provinces. Table S2: Evaluation results of the MaxEnt model generated by ENMeval. AICc, Akaike The akaike information criterion corrected; AUC, The area under the subject curve; Dela.AlCc, AICc the minimum information criterion AICc value; diff.AUC, Average difference between the training and testing AUC; LQHP, Linear features + Quadratic features + Product features + Hinge features; LQPTH, Linear features + Quadratic features + Product features + Threshold features + Hinge features. Table S3: Relative contribution of environmental variables to the final model. Figure S1: ∆AICc values of the Maxent models under different regularization multipliers (RM) and feature combinations (FC); Receiver operating characteristic (ROC) curve; AUC curve (a, Default_AUC; b, ∆AICc values; c, Optimized_AUC). Figure 2. Zones with Model‐Predicted Relative Probability of Occurrence for Tufted Deer in China. HPZ, High model‐predicted relative probability of occurrence zones (0.5–1.0); LPZ, Low model‐predicted relative probability of occurrence zones (0.1–0.25); MPZ, Moderate model‐predicted relative probability of occurrence zones (0.25–0.5); NOT SUM, Total model‐predicted relative probability of occurrence zones under 0.1 threshold. Figure S3: Zones of vacancies in Predicted occurrence probability for Tufted Deer in China. (HPZ_P, High model‐predicted relative probability of occurrence zones within protected areas (0.5–1.0); LPZ_P, Low model‐predicted relative probability of occurrence zones within protected areas (0.1–0.25); MPZ_P, Moderate model‐predicted relative probability of occurrence zones within protected areas (0.25–0.5)). [file ECE3-16-e72850-s001.docx]

**Appendix files**

**Appendix Table 1 The occurrence record of the tufted deer in different provinces**

| Province | Occurrences |
| --- | --- |
| Sichuan | 159 |
| Yunnan | 43 |
| Gansu | 30 |
| Zhejiang | 29 |
| Guangxi | 28 |
| Shaanxi | 27 |
| Guizhou | 22 |
| Hubei | 20 |
| Fujian | 17 |
| Hunan | 13 |
| Jiangxi | 10 |
| Chongqing | 9 |
| Anhui | 8 |
| Guangdong | 6 |
| Xizang | 5 |
| Qinghai | 3 |
| Total | 429 |

**Appendix Table 2 Evaluation results of the MaxEnt model generated by ENMeval.**

| Type | Default | Optimized |
| --- | --- | --- |
| RM | 1 | 1.5 |
| FC | LQHP | LQPTH |
| Delta.AICc | 95.169 | 0.000 |
| diff.AUC | 0.049 | 0.050 |
| AUC | 0.941 | 0.948 |

**Notes**: AICc: Akaike The akaike information criterion corrected; Dela.AlCc: AICc the minimum information criterion AICc value; diff.AUC: Average difference between the training and testing AUC; AUC: The area under the subject curve; LQHP: Linear features + Quadratic features + Product features + Hinge features; LQPTH: Linear features + Quadratic features + Product features + Threshold features + Hinge features.

**Appendix Table 3 Relative contribution of environmental variables to the final model**

| Variable | Percent Contribution (%) | Permutation Importance |
| --- | --- | --- |
| Bio7 | 53.6 | 27.6 |
| Bio12 | 19.2 | 33.9 |
| Bio11 | 6.9 | 5.9 |
| Slope | 5 | 2.6 |
| VFC | 3.5 | 0.9 |
| HFP | 3.4 | 9.9 |
| Altitude | 2.9 | 9.7 |
| Bio15 | 2.2 | 3.5 |
| PD | 1.8 | 1.3 |
| Bio3 | 1.1 | 2.8 |
| Bio19 | 0.3 | 2 |

**Appendix Figure**

**
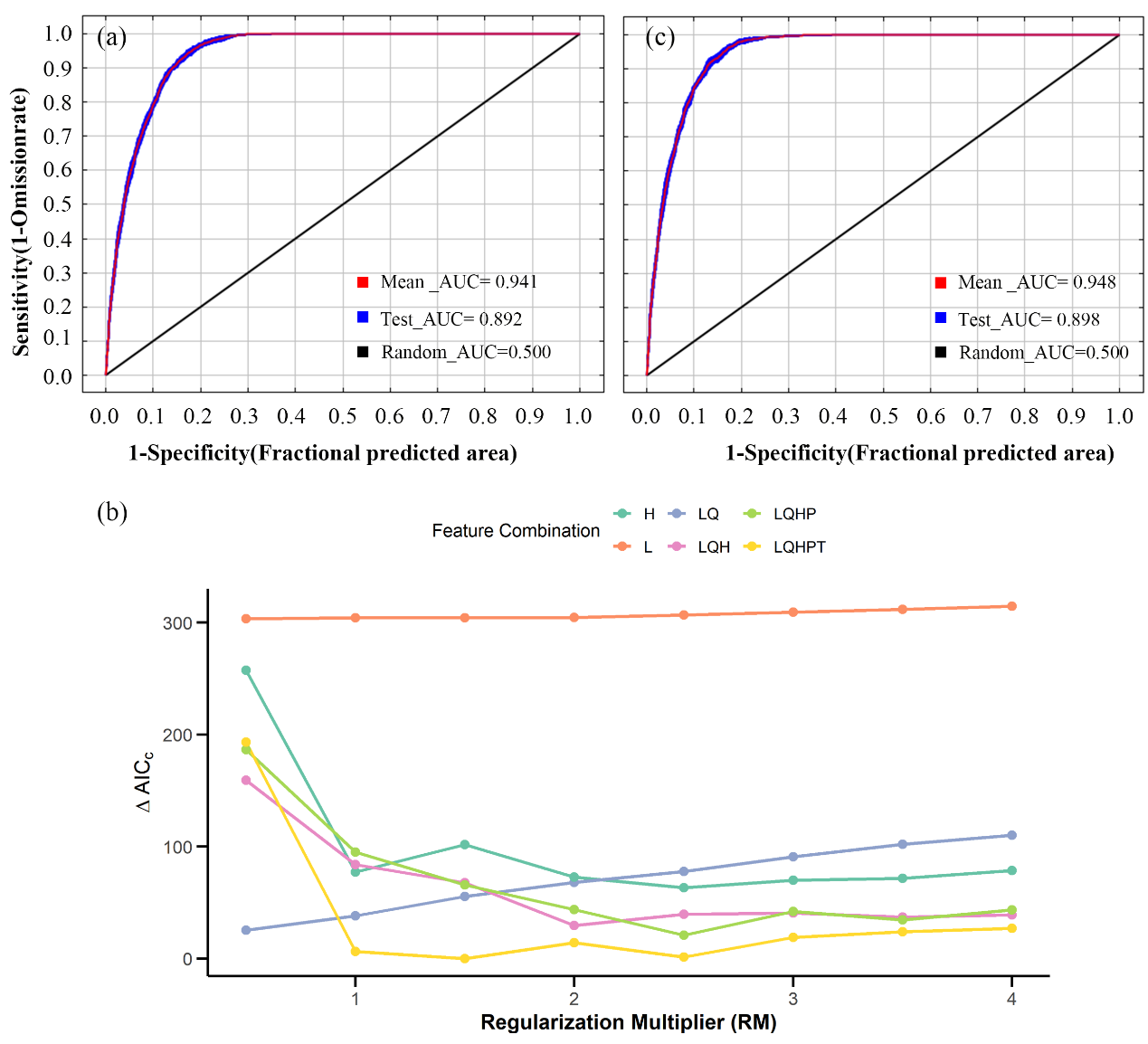
**

**Appendix Figure 1 ∆AICc values of the Maxent models under different regularization multipliers (RM) and feature combinations (FC); Receiver operating characteristic (ROC) curve; AUC curve (a, Default_AUC; b, ∆AICc values; c, Optimized_AUC)**

**
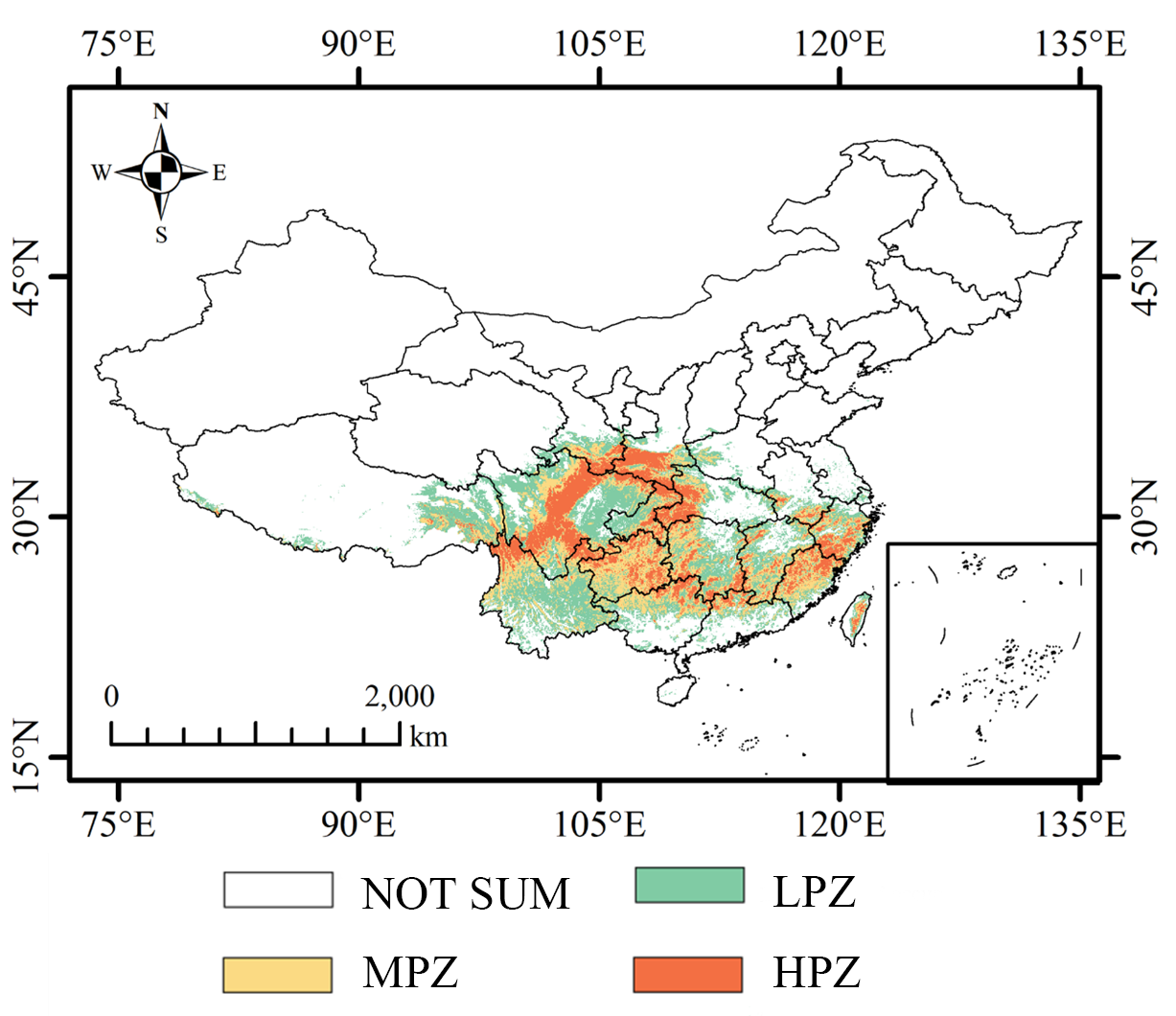
**

**Appendix Figure 2 Zones with Model-Predicted Relative Probability of Occurrence for Tufted Deer in China.** (HPZ: High model-predicted relative probability of occurrence zones (0.5–1.0); MPZ: Moderate model-predicted relative probability of occurrence zones (0.25–0.5); LPZ: Low model-predicted relative probability of occurrence zones (0.1–0.25); NOT SUM: Total model-predicted relative probability of occurrence zones under 0.1 threshold**.**

**
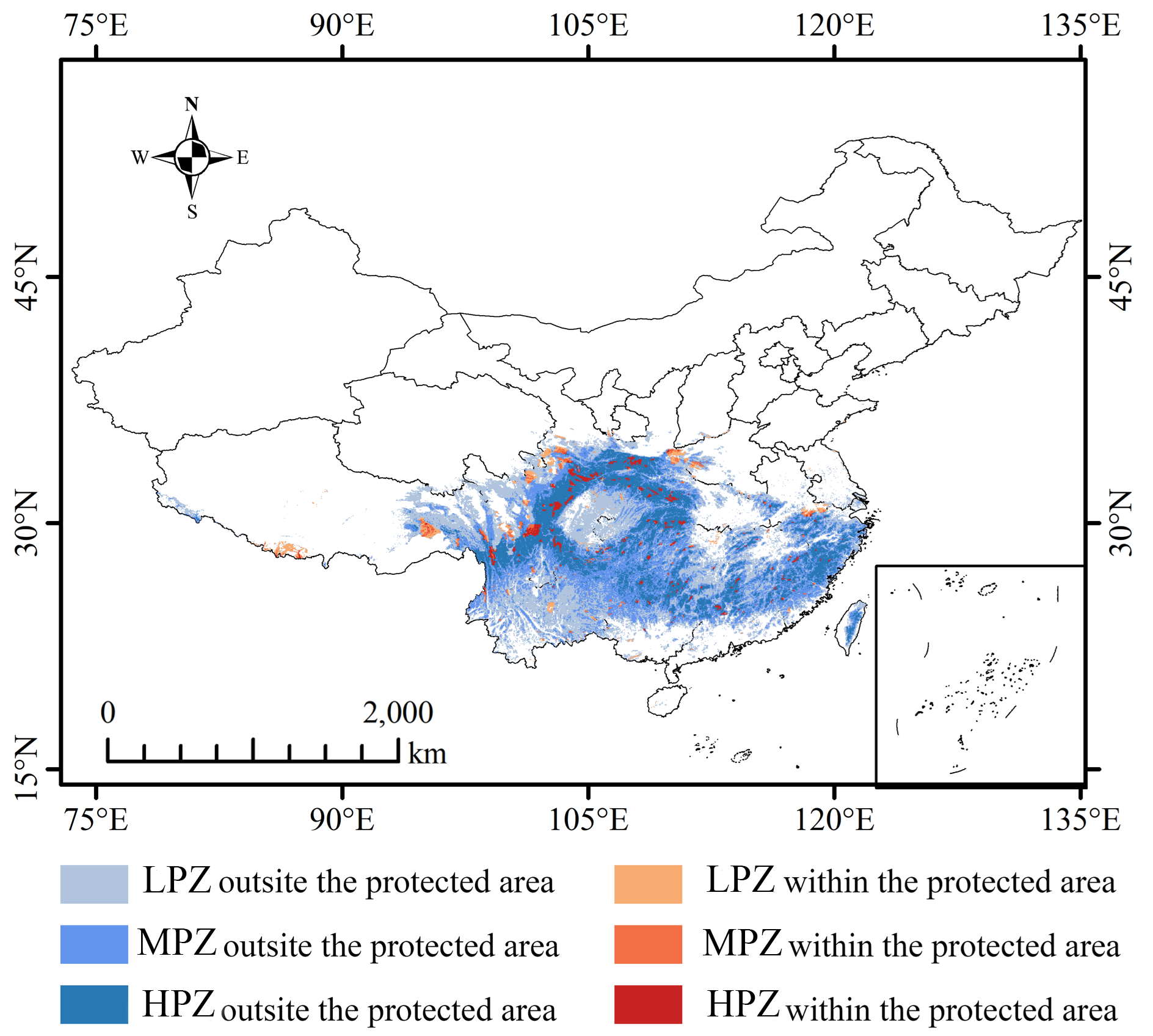
**

**Appendix Figure 3 Zones of vacancies in Predicted occurrence probability for Tufted Deer in China. (**HPZ_P: High model-predicted relative probability of occurrence zones within protected areas (0.5–1.0); MPZ_P: Moderate model-predicted relative probability of occurrence zones within protected areas (0.25–0.5); LPZ_P: Low model-predicted relative probability of occurrence zones within protected areas (0.1–0.25)).
